# Helicteric Acid, Oleanic Acid, and Betulinic Acid, Three Triterpenes from* Helicteres angustifolia* L., Inhibit Proliferation and Induce Apoptosis in HT-29 Colorectal Cancer Cells via Suppressing NF-*κ*B and STAT3 Signaling

**DOI:** 10.1155/2017/5180707

**Published:** 2017-02-26

**Authors:** Dan Su, Yu-qiao Gao, Wei-bo Dai, Ying Hu, Yan-fen Wu, Quan-xi Mei

**Affiliations:** ^1^College of Materials Science and Food Engineering, Zhongshan Institute, University of Electronic Science and Technology of China, Zhongshan, Guangdong 528400, China; ^2^The Zhongshan Affiliated Hospital, Guangzhou University of Chinese Medicine, Zhongshan, Guangdong 528401, China

## Abstract

Colorectal cancer (CRC) is one of the most common malignancies and most frequent cause of cancer death worldwide. The activation of both NF-*κ*B and STAT3 signaling and the crosstalk between them play an important role in colorectal tumor.* Helicteres angustifolia* L. is a type of commonly used Chinese medicinal herb and possesses a wide variety of biological activities. In the present study, we investigate the effects of three triterpenes from* H. angustifolia* (HT) such as helicteric acid (HA), oleanic acid (OA), and betulinic acid (BA), on inhibiting CRC progression. Our results showed that HT extracts could decrease proliferation and induce apoptosis in HT-29 colorectal cancer cells. Moreover, HT extracts could suppress LPS-triggered phosphorylation of IKK, I*κ*B, and NF-*κ*B, attenuate IL-6-induced phosphorylation of JAK2 and STAT3, and suppress the expression of c-Myc, cyclin-D1, and BCL-xL, the downstream gene targets of NF-*κ*B and STAT3. Therefore, HT extracts showed potent therapeutic and antitumor effects on CRC via inhibiting NF-*κ*B and STAT3 signaling.

## 1. Introduction

Colorectal cancer (CRC) is one of the most common malignant cancers worldwide [1–3], in particular in the developed western countries. However, at present its incidence is also increased in the developing countries, especially in China [4]. Although many improvements in chemotherapy and surgical techniques have been made, thus reducing CRC incidence and mortality rates, more than 20% of CRC patients are still associated with poor clinical outcomes [1, 5].

CRC initiation, malignant transformation, and progression are commonly associated with inflammation [6–8]. High levels of inflammatory markers have been usually detected in CRC patients. Interleukin- (IL-) 6 in the serum, for example, is detected in the majority of CRC patients and related to a remarkable high mortality [9, 10]. Recently, NF-*κ*B and STAT3 activation were found to play very critical roles in CRC cell growth, survival, invasion, and migration [11–14].

The effects of natural products from plants have been investigated to a large extent on cancer cell proliferation, survival, invasion, and metastasis due to their bioactivities and diversity of chemical constituents [15–17].* Helicteres angustifolia* L. (Sterculiaceae) (HT), known as “Shan-Zhi-Ma” in Chinese, one of the herbal medicines widely used in traditional medicine in southern China, has been reported to be effective in the treatment of influenza fever, bacterial infections, inflammation, and even cancer [18, 19].

The biological activities of many chemical compounds extracted from HT have been reported [18, 20]. HT and its triterpenes possess anti-inflammatory and antitumor effects in cancer cells, including colorectal tumor HT-29 cell [18, 21]. Triterpenes are considered as HT bioactive chemical components [22]. Methyl helicterate and helicteric acid (HA) are the compounds present in the greatest concentration and have been considered as the characteristic and effective constituents in HT. Our pilot study showed that HT had a significant anti-inflammation activity since it downregulated IL-6 level on 2,4,6-trinitrobenzenesulfonic acid- (TNBS-) induced ulcerative colitis (UC) in mice [23]. Since ILs (especially IL-6) play a crucial role in NF-*κ*B and STAT3 signaling pathways [9, 12], according to our previous findings above we hypothesized that the antitumor activity of triterpenes from HT could be due to their role in both these two pathways and their crosstalk.

In the present study, we demonstrated that helicteric acid (HA), oleanic acid (OA), and betulinic acid (BA), three triterpenes from HT, possessed anti-CRC effects through decreasing inflammation, inhibiting cell proliferation, and promoting apoptosis of HT-29 cells through their action on both NF-*κ*B and STAT3 pathways.

## 2. Materials and Methods

### 2.1. Chemicals

**Figure 1:**
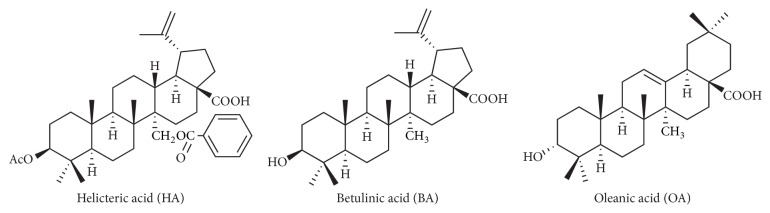
Chemical structures of triterpenes from HT.

In our pilot study methyl helicterate, one of the chemical compounds present in HT, could only be dissolved in DMSO over 5‰, which is a level that could induce significant toxic effects and cell-cycle delay on HT-29 cells. Therefore, helicteric acid (HA), oleanic acid (OA), and betulinic acid (BA) were selected for our experiments and extracted from HT according to our previous study [24]. The chemical structures of HA, OA, and BA are shown in Figure 1.

All the chemicals were dissolved in DMSO to prepare the 10 mM stocks. Recombinant human IL-6 (PeproTech, Rocky Hill, NJ, USA) was dissolved in 1x PBS containing 0.1% BSA to prepare a 10^3^ ng/mL stock. Lipopolysaccharide (LPS, Sigma-Aldrich, Missouri, USA) was dissolved in 1x PBS to prepare a 10^2^ *μ*g/mL stock. All the stocks were stored at −20°C.

### 2.2. Cell Culture

HT-29 carcinoma cell line was kindly provided by the College of Life Science and Technology, Jinan University. Cells were cultured in a 5% CO_2_ atmosphere with 100% humidity at 37°C, in Dulbecco's modified Eagle medium (DMEM) supplemented with 10% fetal calf serum (Gibco, Grand Island, NY, USA), 50 units/mL penicillin, 50 mg/mL streptomycin, and 2 mmol/L L-glutamine. Cells were harvested using 0.25% trypsin (Gibco, Grand Island, NY, USA) and 1 mmol/L EDTA.

### 2.3. Cell Viability Measurement

HT-29 cells were seeded in 96-well plates at a density of 5 × 10^3^ cells/well, in 100 *μ*L cell culture medium per well, and incubated for 24 h. Thereafter, the culture medium was replaced with a fresh one containing drugs (HA and BA at increasing doses such as 1, 5, 10, 25, 50, and 100 *μ*M; OA at 1, 10, 50, 100, 200, and 300 *μ*M), and cells were allowed to grow for another 48 or 72 h. Total cell number was counted and the viability was evaluated using Cell Counting Kit-8 (CCK-8, Dojindo, Tokyo, Japan) according to the kit instructions. CCK-8 (10 *μ*L/well) solution was added and incubated for 2 h, and the absorbance was measured at 450 nm using Multiskan FC microplate photometer (ThermoFisher, Rockford, IL, USA). The results are representative of three independent experiments in triplicate.

### 2.4. Apoptosis Assay

HT-29 cells were seeded in 6-well plates at a density of 5 × 10^4^ cells/well, in 2 mL cell culture per well. Cells were harvested at 48 h and 72 h after the drug treatment (HA and BA at 1, 5, 10, and 25 *μ*M; OA at 1, 10, 50, and 100 *μ*M) and resuspended in cold PBS for analysis. Apoptosis assay was performed using the BD Pharmingen® FITC Annexin V Apoptosis Detection Kit (BD Biosciences, San Jose, CA, USA) according to the manufacturer's instructions. Data were obtained and analyzed using BD FACS Calibur flow cytometry (BD Biosciences).

### 2.5. Measurement of IL-6 Release from HT-29 Cells

HT-29 cells in 6-well plates (2 × 10^5^ cells/well, 2 mL per well) were incubated with drugs (HA and BA at 10 and 25 *μ*M; OA at 50 and 75 *μ*M) for 1 h prior to stimulation with LPS (1 *μ*g/mL) for 24 h. At the end of the experiment, the culture supernatants were collected and centrifuged at 3,000 ×g at 4°C for 10 min to remove any cell debris. IL-6 levels in the media were measured by ELISA kits (Pierce-Endogen, Rockford, IL, USA) according to the manufacturer's instructions.

### 2.6. Western Blot Analysis

HT-29 cells were seeded in 6-well plates at a density of 5 × 10^4^ cells/well in 2 mL cell culture per well. Cells were treated with HT extracts at different concentrations (HA at 5 and 10 *μ*M, BA at 10 *μ*M, and OA at 50 *μ*M) for 2 h prior to stimulation with LPS for 30 min and were harvested for NF-*κ*B signaling determination. Cells were treated with HT extracts at the same concentrations as above for 2 h prior to stimulation with IL-6 for 15 min and were harvested for STAT3 signaling determination. Cells without drug treatment were used as control group. The above analyses were performed in three independent experiments.

Cells of all the above groups were lysed and total proteins were extracted using RIPA lysis buffer (Beyotime, Shanghai, China) plus the PhosStop (Roche, Indianapolis, IN, USA) phosphatase inhibitors and Complete Ultra protease inhibitors (Roche). Equal amounts of protein were used to perform electrophoresis on a 12% SDS-polyacrylamide gel and subsequently transferred to polyvinylidene difluoride (PVDF) membranes (Millipore, Billerica, MA, USA). After blocking with 5% BSA in Tris-buffered saline (TBS) containing 0.1% Tween-20 for 1 h at room temperature, the membranes were incubated overnight at 4°C with the following primary antibodies (1 : 1000): rabbit mAb: phospho-STAT3 (Tyr705), phospho-JAK2 (Y1007/1008), STAT3, JAK2, phospho-NF-*κ*B p65 (Ser536), NF-*κ*B p65, phospho-IKK*α*/*β* (Ser176/180), phospho-I*κ*B*α* (Ser32), and GAPDH; mouse mAb: IKK*α* and I*κ*B*α*. The primary antibodies above were purchased from Cell Signaling Technology (BSN, USA). Membranes were washed three times using Tris-buffered saline Tween-20 (TBST) for 5 min and incubated for 2 h at room temperature with horseradish peroxidase-conjugated secondary antibody (1 : 1,000; Boster, Wuhan, China). After three TBST washes, the target proteins bands were detected by enhancing Pierce ECL Plus (ThermoFisher, Rockford, IL, USA) reagents and exposed to X-ray film (Eastman Kodak, Rochester, NY, USA) for visualization. GAPDH was used as the internal loading control for protein normalization.

### 2.7. Quantitative PCR

HT-29 cells were seeded in 6-well plates at a density of 5 × 10^4^ cells/well in 2 mL cell culture per well. Cells were treated with HT extracts at different concentrations (HA and BA at 1, 10 and 25 *μ*M; OA at 1, 50 and 100 *μ*M). Cells without drug treatment were used as control group.

Total RNA was extracted from cells using Tritrol reagent (Invitrogen, Carlsbad, CA, USA). RNA was reverse-transcribed into cDNA using PrimeScript II RT reagent kit (Takara, Dalian, China) according to the manufacturer's instructions. Real-time quantitative PCR (qPCR) was performed to quantify mRNA levels using the SYBR-Green PCR kit (Roche, Indianapolis, IN, USA) on the LightCycler 480 Real-Time PCR System (Roche). The PCR reaction was carried out as follows: 95°C for 30 sec, 35 cycles at 95°C for 5 sec, and 60°C for 31 sec; and the dissociation stage was as follows: 95°C for 15 sec, 60°C for 1 min, and 95°C for 15 sec. The primers used for qPCR, designed by and purchased from Takara, were the following: cyclin D1 (CCND1), forward: 5′-GTGCATCTACACCGACAACTCC-3′ and reverse: 5′-GTTCCACTTGAGCTTGTTCACC-3′; c-Myc, forward: 5′-GGAGGCTATTCTGCCCATTTG-3′ and reverse: 5′-CGAGGTCATAGTTCCTGTTGGTG-3′; BCL-xL, forward: 5′-CTGGCTCCCATGACCATACTGA-3′ and reverse: 5′-TGAGGCAGCTGAGGCCATAA-3′; GAPDH, forward: 5′-GCACCGTCAAGGCTGAGAAC-3′ and reverse: 5′-TGGTGAAGACGCCAGTGGA-3′. All reactions were performed in triplicate. Relative gene expression was determined using the 2^−ΔΔCT^ method, using GAPDH as the endogenous reference gene.

### 2.8. Statistical Analysis

Statistical analysis was performed using GraphPad Prism Version 5.01. Data are expressed as means ± standard deviation (SD). The significance of the differences between groups was estimated by one-way ANOVA, adjusting for repeated measures with Dunnett's multiple comparison tests. *P* < 0.05 was considered statistically significant.

## 3. Results

### 3.1. HT Extracts Inhibit Proliferation and Induce Apoptosis in HT-29 Cell Line

**Figure 2:**
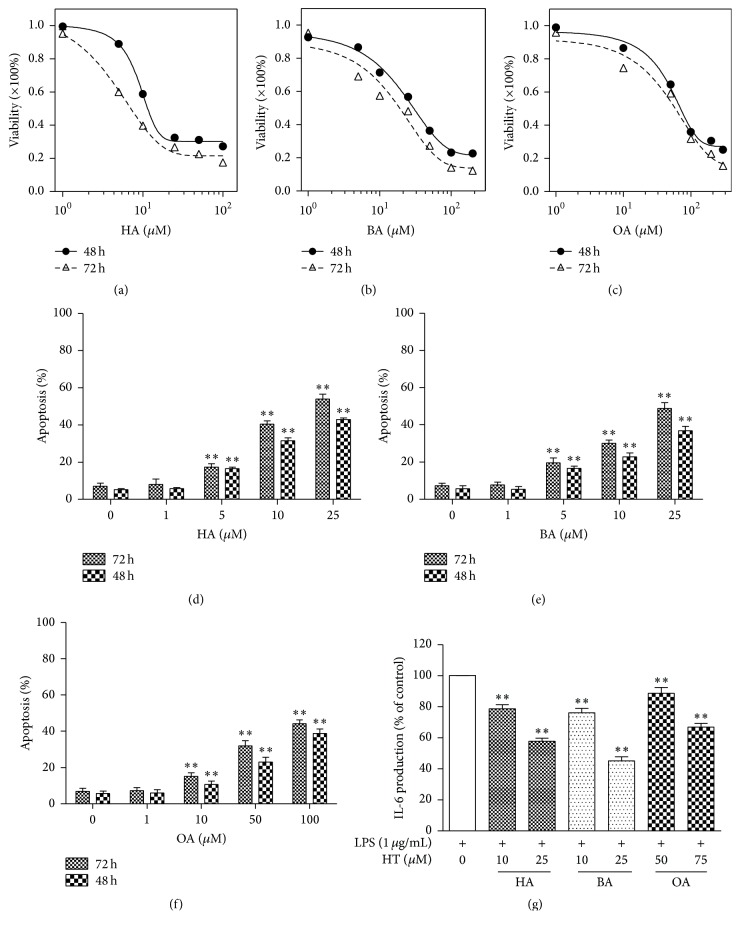
Treatment with HT extracts inhibits cell proliferation and induces apoptosis. (a–c) Cells were treated with HT extracts at the indicated concentrations for 48 or 72 h and CCK-8 cell proliferation assay was performed (*n* = 5/group). (d–f) Cells were treated with HT extracts at the indicated concentrations for 48 of 72 h and the percentage of apoptotic cells was determined by flow cytometry using Annexin V/PI staining (*n* = 5/group). (g) Cells were treated with HT extracts at the indicated concentrations for 1 h prior to stimulation with LPS (1 *μ*g/mL) for 24 h and IL-6 in the supernatants was determined by ELISA. Data are expressed as means ± SD. ^*∗∗*^*P* < 0.01 versus untreated control group.

Our results showed that HT extracts could markedly inhibit HT-29 cells growth in a dose-dependent manner (Figures 2(a)–2(c)). The IC50 at 48 h was ~10 *μ*M for HA, ~10 *μ*M for BA, and ~2 *μ*M for OA (*n* = 5, *P* < 0.05). However, the IC50 at 72 h did not show remarkable differences compared to that at 48 h.

In addition, HT extracts could induce HT-29 cells apoptosis in a dose-dependent manner (Figures 2(d)–2(f)). The apoptotic rate was significantly increased from ~5 *μ*M onward for HA and BA and from ~10 *μ*M onward for OA at 48 or 72 h (*n* = 5, *P* < 0.05) compared with the untreated control cells. The results suggested that HT extracts were capable of inhibiting proliferation and inducing apoptosis in HT-29 tumor cells.

### 3.2. HT Extracts Inhibit IL-6 Release from HT-29 Cell Line

HA at 10 *μ*M and 25 *μ*M represented the concentrations inducing an IL-6 decrease of ~20% and ~40%, respectively, on HT-29 cells; BA at 10 *μ*M and 25 *μ*M, ~25% and ~55%, respectively; OA at 50 *μ*M and 75 *μ*M, ~15% and ~35%, respectively (*n* = 6, *P* < 0.05) (Figure 2(g)).

### 3.3. HT Extracts Inhibit Apoptotic-Related Genes Expression in HT-29 Cell Line

**Figure 3:**
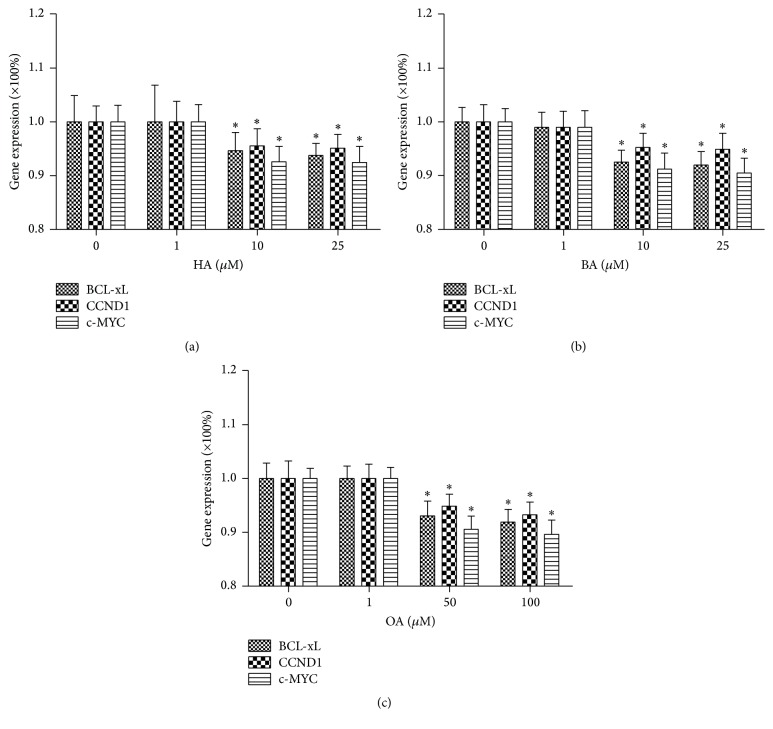
HT extracts inhibit gene expression. (a–c) Cells were treated with HT extracts at the indicated concentrations and quantitative PCR was performed (*n* = 6/group). Data are expressed as means ± SD. ^*∗*^*P* < 0.05 versus untreated control group.

We additionally investigated the effect of triterpenes on genes modulating the proliferation and apoptosis of tumor cells by RT-qPCR. Our results showed that HT extracts significantly blocked c-Myc, CCND1, and BCL-xL gene expression, markedly decreased from ~10 *μ*M onward for HA, from ~10 *μ*M for BA, and from ~50 *μ*M for OA (*n* = 6, *P* < 0.05) (Figures 3(a)–3(c)). The genes above-mentioned are also the downstream genes of NF-*κ*B and STAT3 signaling pathway and they are related to NF-*κ*B and STAT3 phosphorylation.

### 3.4. HT Extracts Inhibit LPS-Induced NF-*κ*B Signaling Pathways

**Figure 4:**
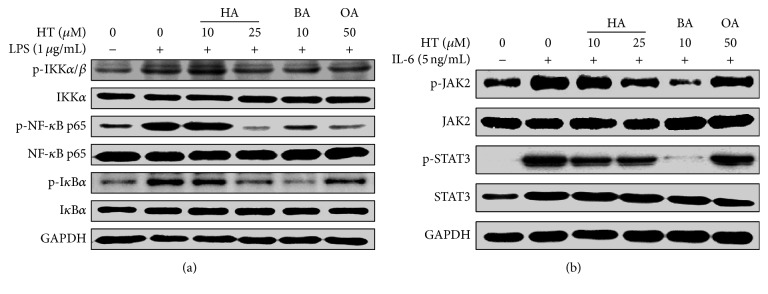
Effects of HT extracts on NF-*κ*B and STAT3. (a) Phosphorylated NF-*κ*B p65 protein in HT-29 cell line was determined by western blot after treatment with HT extracts at the indicated concentrations for 2 h prior to stimulation with LPS for 30 min. (b) Phosphorylated STAT3 protein in HT-29 cell line was determined by western blot after treatment with HT extracts at the indicated concentrations for 2 h prior to stimulation with IL-6 for 15 min. GAPDH was used as the internal control for protein normalization.

HT extracts treatment attenuated IKK/NF-*κ*B pathway in HT-29 cells. The results indicated that HT extracts at high concentration could clearly inhibit LPS-induced IKK, I*κ*B, and NF-*κ*B p65 phosphorylation but did not affect IKK, I*κ*B, and NF-*κ*B p65 protein level in HT-29 cells (Figure 4(a)).

### 3.5. HT Extracts Inhibit IL-6-Induced STAT3 Signaling Pathways

HT extracts attenuated IL-6-induced phosphorylation of JAK/STAT in CRC cell line. However, JAK2 and STAT3 protein level did not change after HT extracts treatment. In addition, JAK2 and STAT3 phosphorylation were significantly raised in HT-29 cells after IL-6 stimulation (*n* = 3) (Figure 4(b)).

## 4. Discussion

CRC is one of the most common human cancers in China and worldwide. The normal colon epithelium switches into malignant tumor due to alterations in specific key genes [9, 25]. In CRC, cancer cells usually become addicted to inflammatory stroma. Then, it progresses as metastasized untreatable cancer. Chronic inflammation may be involved in all steps of CRC development, such as initiation, promotion, and progression, and appears to play a chief role in tumor promotion and progression [12, 26–29].

Tumor microenvironment and its signaling pathways are considered as attractive targets for cancer prevention and therapy. The inflammatory cytokines, such as TNF-*α*, IL-6, and IL-1*β*, and the transcription pathways related to them [30, 31], especially NF-*κ*B and STATs, are considered as potential targets for anti-CRC therapy [11, 13, 32–36].

Herbal phytochemicals consist of a wide variety of biologically active chemical constituents that are ubiquitous in plants, many of which have been reported to have anti-CRC properties [11, 37, 38]. Among them, HT possesses anti-inflammatory effects and potential therapeutic properties against CRC. However, little is known on the effects of HT extracts and their mechanism of action.

An enormous amount of data strongly demonstrated that inhibition of NF-*κ*B signaling could be potentially effective in suppressing inflammation during tumor progression [35, 36]. In colon cancer cells, NF-*κ*B is always constitutively activated and contributes to enhancing proliferation and evasion of apoptosis [13, 39, 40]. In this study, we used LPS-stimulated HT-29 cells as an in vitro CRC model and we observed that pretreatment with HT extracts suppressed LPS-triggered phosphorylation of IKK, I*κ*B, and NF-*κ*B, with HA showing these effects in a dose-dependent manner. These findings suggested that the triterpenes from HT inhibited LPS-induced production of the proinflammatory cytokine IL-6 by blocking, at least in part, NF-*κ*B activation and subsequent transcription.

Recently, many studies showed that STAT3 pathway is involved in inflammation, proliferation, invasion, and migration of tumor cells [6, 32]. Constitutive activation of STAT3 is strongly associated with CRC development [41, 42]. Therefore, suppressing STAT3 activation to inhibit cell proliferation and/or promote apoptosis has been a leading target in the development of anti-CRC treatment. Patients with CRC often exhibit persistently activated STAT3, which could protect cells from apoptotic stimuli and promote cell-cycle progression in colorectal tumors [43]. Thereby these evidences suggest that interfering in the IL-6/JAK/STAT3 pathway could be a new therapeutic strategy for CRC treatment. Our result showed that HT extracts inhibited proliferation and induced apoptosis of HT-29 tumor cell line. We also found that they suppressed IL-6-induced phosphorylation of JAK2 in HT-29 cells, which is in accordance with the decreased phosphorylation of STAT3.

Both NF-*κ*B and STAT3 play central roles in inflammatory cancers, especially in CRC [12, 14]. At present, an enormous amount of data strongly highlighted that inhibition of both NF-*κ*B and STAT3 signaling, which are usually persistently activated in malignant tumors, could be potentially effective in suppressing inflammatory progression in cancers [44]. Our present findings indicated that triterpenes from HT could suppress activation of both IKK/NF-*κ*B and JAK2/STAT3 pathway. Moreover, we found that HT extracts attenuated the expression of c-Myc, cyclin D1, and BCL-xL, which are important genes mediating proliferation and apoptosis, and they are also the downstream gene targets of NF-*κ*B and STAT3. Thus HT extracts exerted their antitumor effects on HT-29 cells by suppressing inflammation and inhibiting proliferation.

However, there are still some important questions that should be elucidated. For example, although our previous study indicated that HT showed less effect on healthy mouse [23], the effects of HT extracts on normal human cells need to be investigated. Furthermore, many recent studies suggest that STAT3 could directly interact with the NF family member RELA in the nucleus, thereby contributing to CRC inflammation and malignant progression [45]. This event could persistently activate STAT3 and NF-*κ*B, thus coregulating many oncogenic and inflammatory genes [46]. Hence, the exact anti-CRC mechanism of this traditional Chinese medicinal herb on NF-*κ*B and STAT3 signaling needs further investigation.

## 5. Conclusion

Overall, our study demonstrated the antitumor activity of triterpenes from HT, indicating that they might be considered as potential therapeutic agents to combat CRC.
